# Clinics at the Buffalo General Hospital by Professor Rochester

**Published:** 1882-01

**Authors:** C. C. Fredericks


### Clinical Reports.

### Clinics at the Buffalo General Hospital by Professor Rochester..

Reported by C. C. Fredericks, M. D.

Case I.—O. B. Scott; entered the hospital Dec. 2, 1881; æt. 74; married; American; patient complains simply of voiding too much urine, in small quantities and frequently; measurement shows the amount of urine passed daily, between three and four quarts, sp. gr. 1030, contains no sugar or albumen. Has had this polyuria for 6 months. An examination of the prostate gland by the finger, per rectum, finds it enlarged and tender.

Case II.—Charles Patterson; entered hospital Nov. 30, 1881; æt. 68; married; American; patient says he is very weak, nauseated, constipated, poor appetite, has vertigo at times, and when he lies down a sensation is felt as of a gale of wind blowing past his ears. He attributes his weakness to excessive excretion of urine, which malady has been gradually increasing for the past six months. His sleep is disturbed by the desire to pass small quantities of urine frequently. Amount of urine per diem, by measurement is about one gallon, sp. gr. 1015, contains no albumen or sugar. Examination of prostate shows it enlarged and tender.

Dr. Rochester thought both of these cases of polyuria due to age, general debility, and most of all, to the condition of the prostate gland in each. He said diabetes mellitus is not so fatal as polyuria when the latter occurs in persons who have led a sedentary life and of great mental activity and strain. These cases he thought not of this kind, and that they would improve with rest, tonics, good diet, and a suppository of ext. belladonna gr. ¼, at bed-time, to relieve the irritability of the bladder and prostate.

Case III.—Thomas M. Spencer; entered hospital Nov. 30, 1881; æt. 64; widower; American. This man has been in the institution twice before during the last six months, under treatment for the same conditions from which he now suffers. Complains of dyspnœa, inability to lie down at night, poor appetite, constipation, anasarca of feet and legs, and general prostration. Examination of urine reveals a small amount of albumen. Area of dullness of liver extends about 2½ inches below the margin of the ribs, and is tender on pressure or percussion. Apex of heart is heard, not seen or felt, faintly between the sixth and seventh ribs, to the left of the nipple. Percussion finds the lower part of the right chest very dull, presumably an effusion of fluid into the right pleura. Exploration with a hypodermic needle gets no fluid, although he has had fluid there at both preceding attacks. Dr. R. advised infusion of digitalis, iodide of potass., salines sufficient to move bowels once daily, tonics, and good food. He spoke of the liability that there is of the physician falling into an error in these cases, viz: That of supposing the effusion into the pleura to be due to the renal or heart difficulties, and not considering that disturbed cardiac action, albumen in the urine, and anasarca is apt to follow from a pleuritic effusion or empyema, and thus be secondary rather than primary.

